# Coronary microvascular resistance comparison of coronary arteries with and without considering vascular diameter: A retrospective, single‐center study

**DOI:** 10.1002/hsr2.1714

**Published:** 2023-12-12

**Authors:** Takahiro Muroya, Hiroaki Kawano, Fumi Yamamoto, Koji Maemura

**Affiliations:** ^1^ Division of Circulatory Sasebo City General Hospital Nagasaki Japan; ^2^ Department of Cardiology Ureshino Medical Center Ureshino Japan; ^3^ Department of Cardiovascular Medicine Nagasaki University Graduate School of Biomedical Sciences Nagasaki Japan

**Keywords:** average peak velocity, coronary blood flow, coronary vessel diameter, hyperemic microvascular resistance index

## Abstract

**Background and Aims:**

Measurement of coronary microvascular resistance (MR) is essential for diagnosing nonocclusive coronary artery ischemia, but whether coronary branches of different diameters can be similarly assessed using hyperemic microvascular resistance index (hMVRI) calculated from average peak velocity (APV) remains unclear. We investigated the relationship between coronary arteries of different diameters and hMVRI.

**Methods:**

Thirty patients with suspected angina pectoris and nonobstructive coronary stenosis with fractional flow reserve >0.8 underwent evaluation of all coronary arteries using a Doppler velocity and pressure‐equipped guidewire. Quantitative coronary angiography (QCA) was used to analyze vessel diameter (D_QCA_). Coronary blood flow (CBF_QCA_) was calculated as πD_QCA_^2^/4 (0.5 × APV) and hMVRI_QCA_ as distal coronary pressure divided by CBF_QCA_ during maximal hyperemia.

**Results:**

The hMVRI was significantly higher for the right coronary artery than for the left anterior descending artery, but no significant differences between arteries were seen for CBF_QCA_ and hMVRI_QCA_. Although the correlation between CBF_QCA_ and APV was weak, CBF_QCA_ divided into three groups according to D_QCA_ showed very strong correlations with APV. Slopes of the straight line between APV and CBF_QCA_ for small‐, middle‐, and large‐diameter groups were 0.48, 0.30, and 0.21, respectively, with slope decreasing as diameter increased.

**Conclusions:**

Comparative evaluation of MR in coronary branches with varying vessel diameters requires vessel diameter to be accounted for.

## 1 INTRODUCTION

Although angina pectoris affects approximately 112 million people globally, up to 70% of patients undergoing invasive angiography do not have obstructive coronary artery disease. Instead, the main cause is ischemia with no obstructive coronary arteries (INOCA), which can result from heterogeneous mechanisms, including coronary vasospasm and microvascular dysfunction, and is not a benign condition.^1^ Since no currently available modalities allow direct visualization of the human coronary microcirculation in vivo, microvascular evaluation relies on the measurement of parameters that reflect the functional status, such as coronary blood flow (CBF), coronary flow reserve, and microvascular resistance using a diagnostic guidewire. Both microvascular and epicardial vasospastic angina are then assessed with the acetylcholine test. Lifestyle modification, risk factor management, and anti‐anginal medications have been proposed for the management of INOCA. However, studies of therapies to improve coronary microvascular dysfunction (CMD) have been small and heterogeneous in both design and methodology, and evidence‐based treatments for CMD are not currently available. An updated standardization of criteria for microvascular angina (MVA) in patients presenting with angina pectoris or ischemia‐like symptoms in the absence of flow‐limiting coronary artery disease was proposed by the COVADIS group, with abnormal coronary microvascular resistance indices among the leading diagnostic parameters. Although these parameters can be investigated using the index of microcirculatory resistance (IMR) or hyperemic microvascular resistance index (hMVRI), evaluation in the catheterization laboratory has become easier in recent years and guidelines recommend IMR and hMVRI measurements to investigate INOCA. Many reports^1^, ^2^, ^3^, ^4^, ^5^, ^6^, ^7^, ^8^, ^9^, ^10^, ^11^, ^12^, ^13^, ^14^, ^15^, ^16^, ^17^, ^18^, ^19^, ^20^ have already examined the usefulness and pitfalls of guidewires in the examination of IMR or hMVRI, but which coronary artery should be targeted for evaluation in INOCA remains a vexing question. The left anterior descending artery (LAD) is the preferred target vessel in many studies, reflecting its myocardial mass and coronary arterial predominance, and much of the evidence for physiological assessments in INOCA has been obtained from investigations using the LAD.^7^, ^8^, ^9^ On the flip side, when the LAD cannot be used for technical reasons, use of the left circumflex artery (LCX) is recommended, followed by the right coronary artery (RCA). The reason for this may be that previous reports have shown that hMVRI in the RCA is significantly higher than in other coronary branches.^14^, ^15^ Average peak velocity (APV) is used instead of CBF for coronary microvascular resistance measurement, because IMR and hMVRI are used for simple measurements in the cardiac catheterization laboratory. However, whether coronary branches of widely varying diameters can be evaluated in the same manner using APV in humans remains highly questionable.

To determine whether differences in coronary microcirculatory resistance vary according to vessel size at the site of measurement of the coronary branch, we studied cases in which all coronary branches in the same patient were measured.

## 2 MATERIALS AND METHODS

### 2.1 Patient population

We evaluated patients with clinical suspicion of stable angina pectoris based on the presence of chest pain or chest discomfort. Evaluations were performed using elective coronary angiography and physiological assessments between September 2010 and May 2016. A total of 203 patients with fractional flow reserve (FFR) > 0.8 in at least one coronary branch were evaluated. Patients with hypertrophic cardiomyopathy (*n* = 6), moderate‐to‐severe aortic valve stenosis disease (*n* = 3), coronary artery bypass graft surgery (*n* = 1), atrial fibrillation (*n* = 6), cardiac pacemaker insertion (*n* = 5), recent myocardial infarction (<6 weeks before screening) (*n* = 13), visible collateral development to the perfusion territory of interest (*n* = 3) or left ventricular ejection fraction <50% (*n* = 6) were excluded. As a result, we assessed 281 coronary arteries with FFR > 0.8 in 160 patients (Group A), comprising 121 LADs, 83 LCXs, and 77 RCAs.

Of these, 30 patients (Group B) had all three coronary branches available for examination and an FFR > 0.8. Coronary microvascular resistance between coronary branches was then examined separately using both conventional indices and indices that considered vessel size using quantitative coronary angiography (QCA). All patients provided written informed consent for participation. The Ethics Committee at Ureshino Medical Center approved the protocol for the present study (approval no. 10‐10), which was performed in accordance with the principles laid out in the Declaration of Helsinki.

### 2.2 Cardiac catheterization

**Figure 1:**
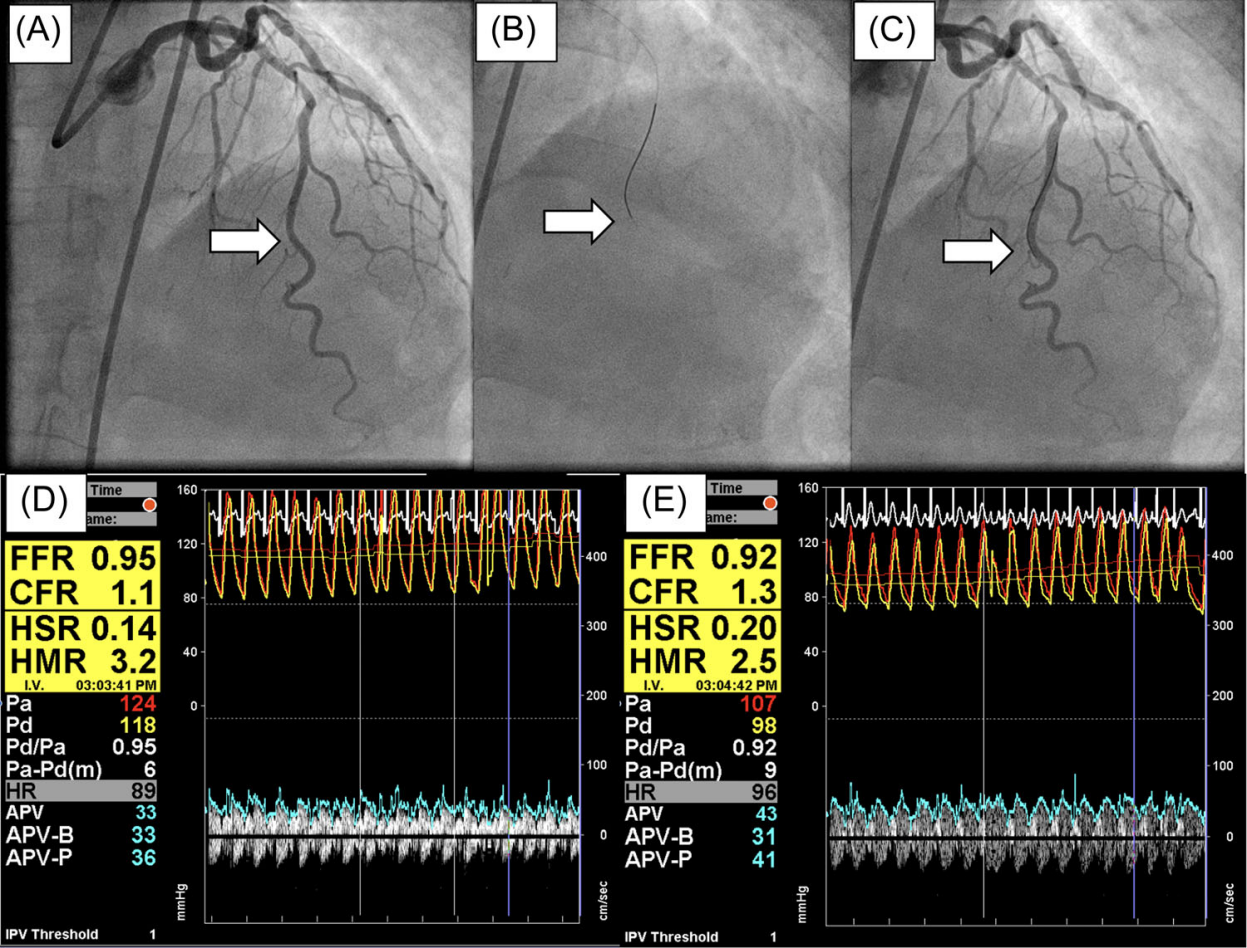
Calculation of coronary physiological values. Hyperemic microvascular resistance index (hMVRI) was calculated as distal coronary pressure (Pd) divided by distal average peak velocity (APV) at maximal hyperemia. Vessel diameter was analyzed by quantitative coronary angiography (QCA) at the site of blood flow measurement by wire (white arrow). QCA was performed with a validated densitometric analysis system (Pie Medical Imaging B.V, Maastricht, the Netherlands). Coronary blood flow (CBF) was calculated as πD^2^/4 (0.5 × APV), where D is vessel diameter. CBF as measured by QCA is described as CBF_QCA_, then hMVRI_QCA_ is calculated as Pd divided by CBF_QCA_ during maximal hyperemia. As CBF_QCA_ will be overestimated, hMVRI_QCA_ will be higher than the original HMR. (A) Coronary angiography. (B) Insertion of the Doppler flow guidewire into the left anterior descending artery. (C) Final position of the Doppler flow guidewire as confirmed by contrast injection. (D) Physiological measurements at rest. (E) Physiological measurements under hyperemic conditions. CFR, coronary flow reserve; HMR, hyperemic microvascular resistance; HSR, hyperemic stenosis resistance.

After coronary angiography, aortic pressure was measured via a 5‐ or 6‐Fr guiding catheter placed in the coronary ostium via a radial or femoral approach. Intracoronary pressure and coronary flow velocity were measured with a 0.014″ Doppler velocity and pressure‐equipped guidewire (Volcano Corp), then the sensor was placed at a site more distal than the distal half of the coronary artery. Hyperemia was induced by papaverine hydrochloride delivered into the coronary artery (12 mg into the left coronary artery and 8 mg into the RCA within 15 s), then blood pressure was recorded at 20 s after the end of administration. FFR was defined as the ratio of mean distal coronary pressure (Pd) to mean aortic pressure (Pa) in the target vessel beyond the lesion during maximal hyperemia. hMVRI was calculated as Pd divided by distal APV during maximal hyperemia. In the present study, those coronary arteries showing FFR > 0.8 were defined as non‐obstructive coronary arteries unaffected by collateral effects,^5^ and were investigated. The estimation of volumetric flow from Doppler flow velocity also incorporates vessel diameter. Volumetric CBF should always be calculated, using QCA to estimate epicardial diameter.^6^, ^8^ Vessel diameter as analyzed by QCA (D_QCA_) was measured at the site of the wire where blood flow was measured (Figure 1). QCA was performed using a validated densitometric analysis system (Pie Medical Imaging B.V., Maastricht, the Netherlands). CBF (CBF_QCA_) was calculated as πD_QCA_^2^/4 (0.5 × APV) (**D**_**QCA**_, **vessel diameter**), then hMVRI_QCA_ was calculated as Pd divided by CBF_QCA_ during maximal hyperemia.

In these patients, CMD was evaluated using modified criteria from previous reports^2^, ^3^, ^4^, ^5^, ^6^, ^7^, ^8^, ^9^, ^10^, ^11^, ^12^, ^13^ for the diagnosis of MVA.

Coronary physiological values were calculated as follows:

$$\begin{array}{l} \text{FFR}=\text{Pd}/\text{Pa}\;\text{during}\;\text{hyperemia}\\ \text{hMVRI}\;\left(\text{mmHg}/\text{cm}/s\right)=Pd/\text{APV}\;\text{during}\;\text{hyperemia}\\ {\text{CBF}}_{\text{QCA}}\left(\text{mL}/\min\right)={\pi D}_{\text{QCA}}^{2}/4\;\left(0.5\times \text{APV}\right)\;\left(D_{\text{QCA}},\text{vessel}\;\text{diameter}\;\text{using}\;\text{QCA}\right)\\ {\text{hMVRI}}_{\text{QCA}}\left(\text{mmHg}/\text{mL}/\min\right)=\text{Pd}/{\text{CBF}}_{QCA}\;\text{during}\;\text{hyperemia}. \end{array}$$

### 2.3 Statistical analysis

All data are expressed as median ± standard deviation or the number and percentage of patients. Non‐parametric paired Wilcoxon analysis testing was used to test for associations of intra‐coronary physiological values, vessel diameter and CBF_QCA_ between the LAD, LCX, and RCA. Associations between APV and CBF_QCA_ in Group B, a small vessel diameter group, middle diameter group, large diameter group, LAD, LCX, and RCA were evaluated using univariate linear regression analysis. Values of *p* < 0.05 were considered statistically significant. Data were statistically analyzed using JMP version 10 software (SAS Institute Inc.).

## 3 RESULTS

### 3.1 Patient characteristics

Patient characteristics in Groups A and B are presented in Table 1. Median age was 69.0 years in Group A and 74 years in Group B. Men comprised 113 of the 160 patients (70.6%) in Group A and 23 of the 30 patients (76.7%) in Group B. No significant differences in patient characteristics were seen between Groups A and B, except for HbA1c. HbA1c was significantly lower in Group B than in Group A (*p* = 0.040), but no significant difference in the incidence of diabetes mellitus was identified (*p* = 0.301).

**Table 1:** **Patient characteristics in Groups A and B**.

Variable	Group A (*n* = 160)	Group B (*n* = 30)	*p* value
**General risk factors**			
Age, year	69.0 ± 9.6	74.0 ± 10.3	0.272
Male sex, *n* (%)	113 (70.6)	23 (76.7)	0.501
Body mass index, kg/m^2^	24.5 ± 3.9	24.5 ± 3.9	0.428
**Cardiovascular risk factors**			
Hypertension, *n* (%)	112 (70)	18 (60.0)	0.280
Dyslipidemia, *n* (%)	110 (68.8)	17 (56.7)	0.197
Diabetes mellitus, *n* (%)	64 (40)	9 (30.0)	0.301
Current smoker, *n* (%)	43 (26.9)	4 (13.3)	0.115
**Laboratory findings**			
HDL, mg/dL	53 ± 16	52 ± 15	0.900
LDL, mg/dL	101 ± 31	97 ± 29	0.296
TG, mg/dL	113 ± 93	97.5 ± 93	0.443
Cr, mg/dL	0.79 ± 0.78	0.81 ± 0.84	0.471
HbA1c, %	5.7 ± 1.1	5.4 ± 1.0	0.040
**CAD**			
Previous MI, *n* (%)	34 (21.3)	6 (20.0)	0.878
Previous PCI, *n* (%)	85 (53.1)	20 (66.7)	0.171
**Medications**			
β‐blocker, *n* (%)	21 (13.1)	4 (13.3)	0.975
Calcium channel blocker, *n* (%)	84 (52.5)	13 (43.3)	0.357
ARB or ACE inhibitor, *n* (%)	77 (48.1)	11 (36.7)	0.248
Statin, *n* (%)	98 (61.3)	17 (56.7)	0.637
Diuretic, *n* (%)	7 (4.4)	1 (3.3)	0.794
**UCG findings**			
LVDd, mm	47.2 ± 3.7	46.9 ± 3.1	0.739
LVDs, mm	29.8 ± 3.8	29.0 ± 3.4	0.373
LVEF, %	66.9 ± 6.5	67.7 ± 7.1	0.301

*Note*: Values are presented as *n* (%) or median ± standard deviation.

Abbreviations: ACE, angiotensin‐converting enzyme inhibitor; ARB, angiotensin receptor blocker; Cr, creatinine; HbA1c, hemoglobin A1c; HDL, high‐density lipoprotein cholesterol; LDL, low‐density lipoprotein cholesterol; LVDd, left ventricular end‐diastolic diameter; LVDs, left ventricular end‐systolic diameter; LVEF, left ventricular ejection fraction; MI, myocardial infarction; PCI, percutaneous coronary intervention; TG, triglycerides; UCG, ultrasonic echocardiography.

### 3.2 Relationship between coronary physiological assessments and coronary artery branches

**Figure 2:**
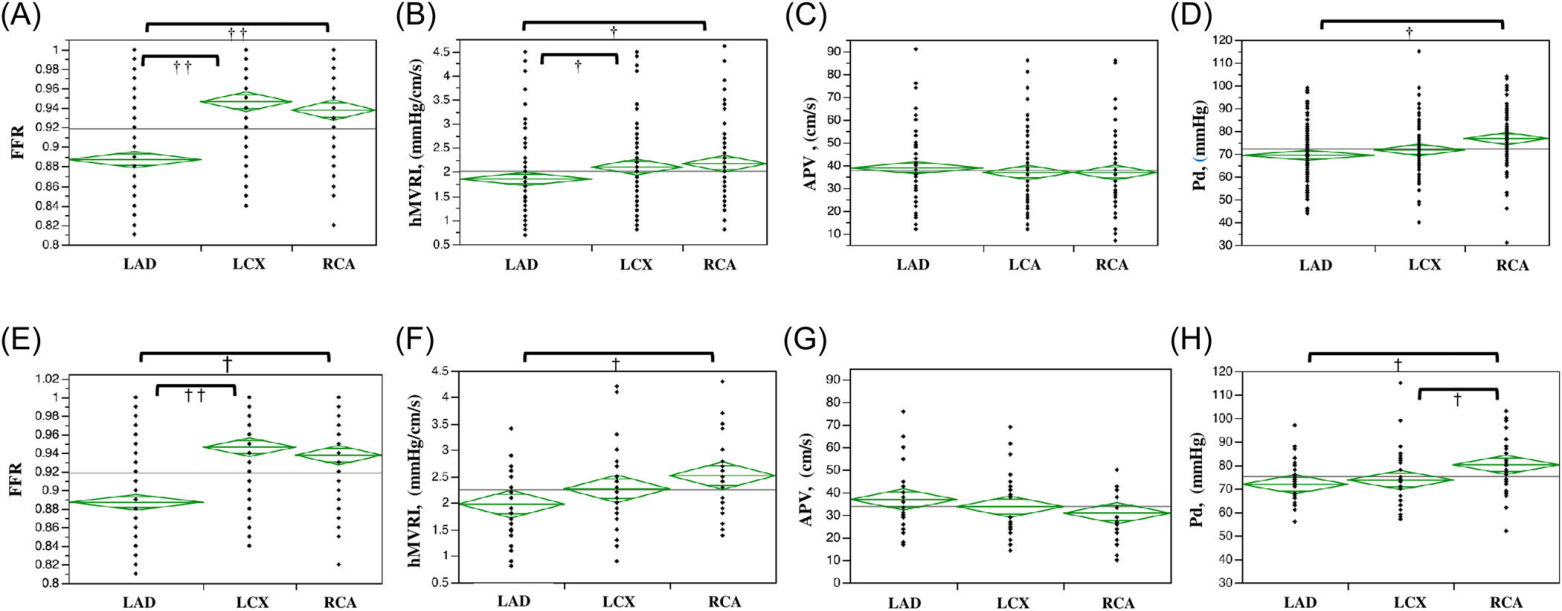
Evaluation of FFR, hMVRI, APV, and Pd in Groups A and B. In comparisons between LAD, LCX, and RCA, significant differences in FFR, hMVRI, and Pd are seen in Group A (A–D) and Group B (E–H). (A) FFR is significantly lower in the LAD than in the LCX or RCA (*p* < 0.001) in Group A. (B) hMVRI is significantly lower in the LAD than in the LCX or RCA (*p* = 0.003) in Group A. (C) No significant differences in APV are seen between the three coronary branches in Group A. (D) Pd is significantly lower in the LAD than in the RCA (*p* < 0.001) in Group A. (E) FFR is significantly lower in the LAD than in the LCX or RCA (*p* < 0.001) in Group B. (F) hMVRI is significantly lower in the LAD than in the RCA (*p* = 0.019) in Group B. (G) No significant differences in APV are seen between the three coronary branches in Group B. (H) Pd is significantly lower in the LAD and LCX than in the RCA (*p* = 0.004) in Group B. ^†^ *p* < 0.05; ^††^ *p* < 0.001. FFR, fractional flow reserve; hMVRI, hyperemic microvascular resistance index; LAD, left anterior descending coronary artery; LCX, left circumflex; Pd, mean distal coronary pressure; RCA, right coronary artery.

Coronary physiological assessments of Groups A and B are presented in Table 2 and Figure 2, respectively. Median values of FFR, hMVRI, APV, and Pd in Group A were 0.92, 1.9, 37, and 72 mmHg/cm/s, respectively. Median values of FFR, hMVRI, APV, and Pd in Group B were 0.94, 1.9, 29, and 74 mmHg/cm/s, respectively.

**Table 2:** Coronary physiological assessments by Doppler and pressure sensor‐equipped guide‐wire in Groups A and B.

Variable	Group A (*n* = 281)	LAD (*n* = 121)	LCX (*n* = 83)	RCA (*n* = 77)	*p* value	Group B (*n* = 90)	LAD (*n* = 30)	LCX (*n* = 30)	RCA (*n* = 30)	*p* value
FFR	0.92 ± 0.05	0.88 ± 0.05	0.96 ± 0.05	0.94 ± 0.05	<0.0001	0.94 ± 0.05	0.90 ± 0.04	0.97 ± 0.04	0.95 ± 0.03	<0.0001
hMVRI, mmHg/cm/s	1.9 ± 0.8	1.7 ± 0.7	2.0 ± 0.8	2.0 ± 0.8	0.003	1.9 ± 0.9	1.9 ± 0.7	2.2 ± 0.8	2.5 ± 0.7	0.019
APV, cm/s	37 ± 15	38 ± 15	36 ± 15	36 ± 15	0.381	29 ± 13	33 ± 14	29 ± 13	29 ± 11	0.332
Pd, mmHg	72 ± 12	70 ± 12	71 ± 12	77 ± 13	<0.0001	74å ± 12	72 ± 10	72 ± 13	79 ± 11	0.004

*Note*: Values are presented as *n* (%) or median ± standard deviation.

Abbreviations: APV, average peak velocity; FFR, fractional flow reserve; hMVRI, hyperemic microvascular resistance index; LAD, left anterior descending coronary artery; LCX, left circumflex; Pd, mean distal coronary pressure; RCA, right coronary artery.

In terms of the relationships between LAD, LCX, and RCA, similarly significant differences in FFR, hMVRI and Pd were seen in Groups A and B (Table 2 and Figure 2). In both Groups A and B, hMVRI was significantly higher for the RCA than for the LAD.

### 3.3 Relationships between D_QCA_, CBF_QCA_, and hMVRI_QCA_ of coronary artery branches in Group B

**Figure 3:**
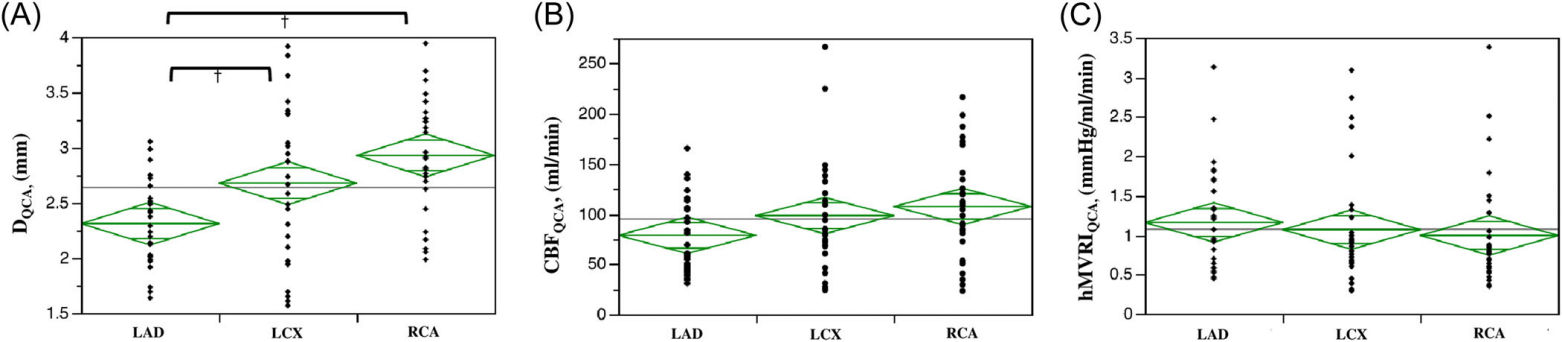
D_QCA_, CBF_QCA_, and HMR_QCA_ corrected for vessel diameter by QCA. (A) D_QCA_ in the LAD, LCX, and RCA are 2.27, 2.81, and 2.91 mm, respectively. D_QCA_ is significantly larger in the LCX and RCA than in the LAD (LCX, *p* = 0.029; RCA, *p* < 0.001), but no significant difference is seen between the LCX and RCA (*p* = 0.152). (B, C) No significant differences in CBF_QCA_ or hMVRI_QCA_ are seen between LAD, LCX, and RCA. ^†^ *p* < 0.05; ^††^ *p* < 0.001. FFR, fractional flow reserve; hMVRI, hyperemic microvascular resistance index; LAD, left anterior descending coronary artery; LCX, left circumflex; Pd, mean distal coronary pressure; RCA, right coronary artery.

D_QCA_ was analyzed by QCA at the site of blood flow measurement by diagnostic wire in Group B, with hMVRI re‐evaluated as hMVRI_QCA_ and CBF as CBF_QCA_. D_QCA_ of the LAD, LCX, and RCA was 2.27, 2.81, and 2.91 mm, respectively; D_QCA_ was significantly larger for the LCX and RCA than for the LAD (LCX, *p* = 0.029; RCA, *p* < 0.001), but no significant difference was seen between D_QCA_ of the LCX and RCA (*p* = 0.152) (Table 3 and Figure 3A). Unlike the results examined in the APV, no significant differences between LAD, LCX, or RCA were seen for CBF_QCA_ or hMVRI_QCA_ (Table 3 and Figure 3B,C).

**Table 3:** Re‐evaluation of Group B using hMVRI_QCA_ and CBF_QCA_.

Variable	LAD	LCX	RCA	*p* value
D_QCA_, mm	2.27 ± 0.39	2.81 ± 0.67	2.91 ± 0.51	0.0002
CBF_QCA_, mL/min	65.0 ± 37.7	82.4 ± 59.1	106.7 ± 50.9	0.100
hMVRI_QCA_, mmHg/mL/min	1.04 ± 0.69	0.87 ± 0.74	0.78 ± 0.69	0.384

*Note*: Values are presented as *n* (%) or median ± standard deviation.

Abbreviations: CBF, coronary blood flow; D, diameter; hMVRI, hyperemic microvascular resistance index; LAD, left anterior descending coronary artery; LCX, left circumflex; RCA, right coronary artery.

### 3.4 APV and CBF_QCA_ for small D_QCA_, middle D_QCA_, and large D_QCA_ in Group B

**Figure 4:**
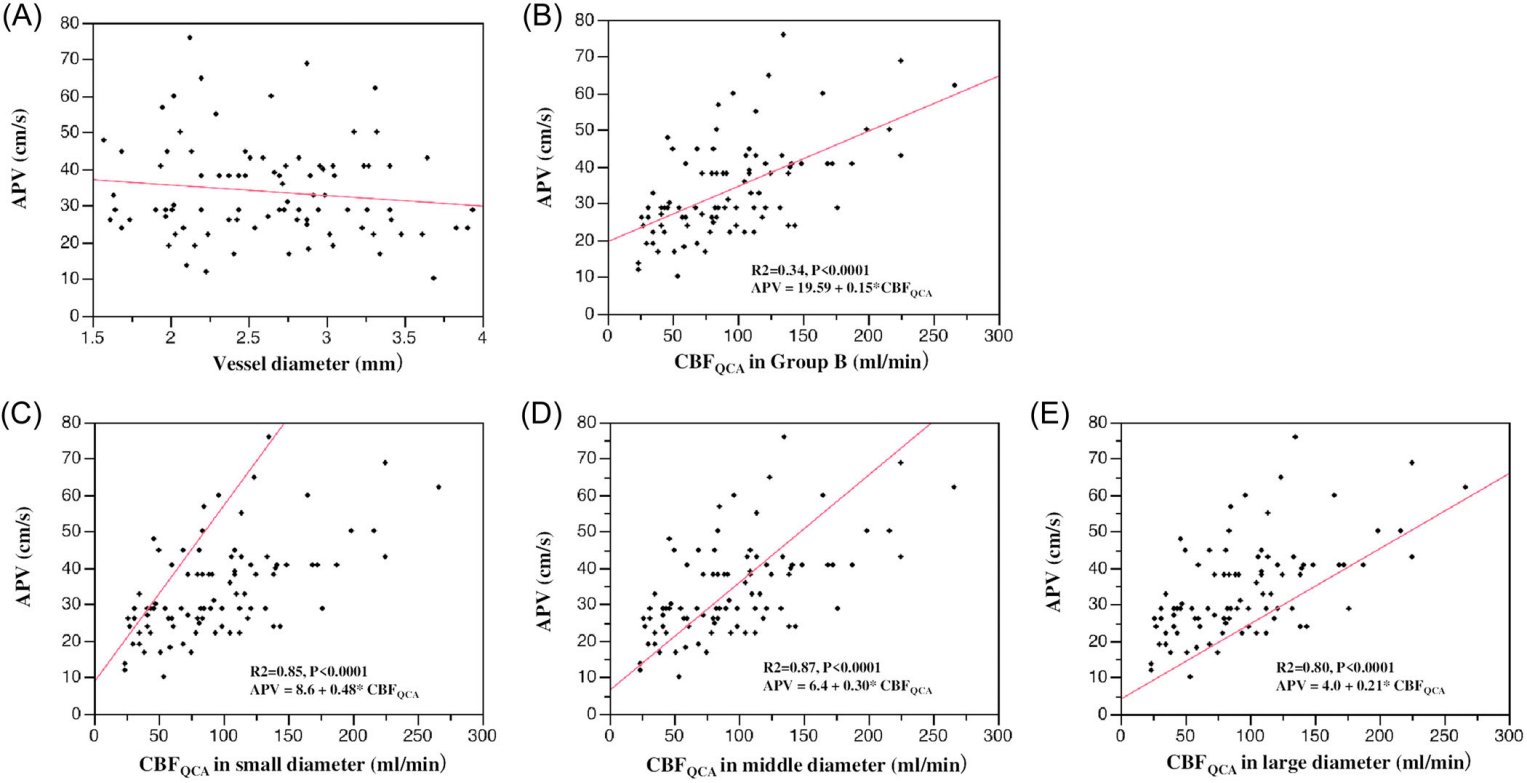
Correlation between APV and CBF_QCA_ in Group B. (A) No collinearity between D_QCA_ and APV is seen in scattergrams (*p* = 0.225). (B) CBF_QCA_ and APV in Group B show a weak correlation (*p* < 0.001, *R* ^2^ = 0.34; APV = 19.59 + 0.15 × CBF_QCA_). We divided Group B into three equal groups according to the D_QCA_: small diameter group (C), 1.57–2.29 mm; middle diameter group (D), 2.31–2.9 mm; and large diameter group (E), 2.92–3.94 mm. (C–E). The correlations between CBF_QCA_ and APV in the small, middle and large diameter groups are strong (small diameter group: *R* ^2^ = 0.85, *p* < 0.001, APV = 8.6 + 0.48 × CBF_QCA_; middle diameter group: *R* ^2^ = 0.87, *p* < 0.001, APV = 6.4 + 0.30 × CBF_QCA_; large diameter group: *R* ^2^ = 0.80, *p* < 0.001, APV = 4.0 + 0.21 × CBF_QCA_). The slope of the straight line between APV and CBF_QCA_ for small, middle, and large diameters is 0.48, 0.30, and 0,21, respectively, decreasing as diameter increases.

We found no collinearity between DQCA and APV in scattergrams (Figure 4A). We then examined the relationship between CBF_QCA_ and APV in Group B, but the correlation was weak (*p* < 0.001, *R* ^2^ = 0.34) (Figure 4B). We further divided vessels into three equal groups according to D_QCA_: small diameter group, 1.57–2.29 mm; middle diameter group, 2.31–2.9 mm; and large diameter group, 2.92–3.94 mm. Associations between APV and CBF_QCA_ were assessed for each group, revealing that CBF_QCA_ in the three groups correlated strongly with APV (small diameter group: *R* ^2^ = 0.85, *p* < 0.001; middle diameter group: *R* ^2^ = 0.87, *p* < 0.001; large diameter group: *R* ^2^ = 0.80, *p* < 0.001) (Figure 4C–E). The slope of the straight line between APV and CBF_QCA_ for small, middle, and large diameter groups was 0.48, 0.30, and 0.21, respectively, with slope decreasing as diameter increased.

## 4 DISCUSSION

The present study examined whether differences exist in microcirculatory resistance in human coronary arteries of different sizes. The main findings were as follows. First, as reported previously, hMVRI was significantly higher for the RCA than for the LAD in Groups A and B. Second, unlike the results for the APV, no significant differences in hMVRI_QCA_ as calculated using D_QCA_ were seen between the LAD, LCX, and RCA. Moreover, the correlation between CBF_QCA_ and APV was weak. However, CBF_QCA_ in patients divided into three groups according to D_QCA_ showed a very strong correlation with APV.

### 4.1 Significantly higher hMVRI for the RCA than for the LAD

In the present study, hMVRI was significantly higher for the RCA than for the LAD in both Groups A and B. Murai et al.^14^ reported that measurement at the RCA site, but not LAD or LCX, and hypertension were independent predictors of an increased IMR, according to the results for IMR in 131 arteries from 104 patients with intermediate obstructive coronary artery lesions. Similarly, Lee et al.^15^ reported that 1096 patients with 1452 coronary arteries enrolled from 8 centers in 5 countries were analyzed by IMR, and predictors of high IMR included previous myocardial infarction, measurement at the RCA site, female sex, and obesity. IMR and hMVRI derive microvascular resistance from simultaneous distal coronary artery measurements of pressure and flow during hyperemia using intra‐coronary guidewires. IMR estimates flow with thermodilution,^3^ whereas hMVRI incorporates Doppler flow velocity.^16^ Both indices have separately been shown to predict infarct size,^17^, ^18^ microvascular obstruction,^18^ regional wall motion,^17^ and adverse left ventricular remodeling. Williams et al.^19^ reported that the correlation between independent invasive and noninvasive measurements of microvascular function was better with hMVRI than with IMR. However, neither of these invasive measures of microvascular resistance consider vessel size.

### 4.2 Relevance of vessel diameter in evaluating CBF and hMVRI

Unlike the results for APV, no significant difference in hMVRI_QCA_ was found for LAD, LCX, or RCA, respectively, when examined using CBF_QCA_. Furthermore, the correlation between APV and CBF_QCA_ was weak. Quantitative measurement of volumetric CBF (in milliliters per minute) during catheterization in humans is not currently possible. In evaluating CBF, a method that accounts for vessel size by QCA has been reported in addition to the semi‐quantitative method using APV. Perera et al.^8^ evaluated angina and non‐obstructive coronary artery disease by calculating epicardial diameter using QCA and calculating CBF for the purpose of evaluating endothelial‐dependent microvascular function by acetylcholine.

Although no collinearity was evident between D_QCA_ and APV in scattergrams, vessel size as divided into three groups allowed identification of a strong correlation between CBF_QCA_ and APV. According to Doucette et al.,^6^ APV showed a very strong linear correlation with CBF in small‐sized straight tubes in vitro and in normal LCX in dogs, but slope decreased as diameter increased. Similarly, in the present study, the slope of the straight line between APV and CBF_QCA_ for small, medium, and large diameters in humans was 0.48, 0.30, and 0.21, respectively, decreasing as diameter increased. Moreover, we assessed associations between APV and CBF_QCA_ for coronary artery branches. The LAD is the preferred target vessel in many studies, reflecting its myocardial mass and coronary arterial predominance, and much of the evidence for physiological assessments in INOCA has been obtained using the LAD.^7^, ^8^, ^9^ On the flip side, when the LAD cannot be used for technical reasons, use of the LCX is recommended, followed by the RCA. Nonetheless, RCAs have been documented to exhibit considerably greater resistance indices than LADs and LCXs when measured through conventional means. Consequently, utilizing the same measurement methodology for RCAs as that for LADs poses a predicament. However, adopting resistance indices that account for vessel diameter is anticipated to yield decreased inaccuracies. Furthermore, measuring indices of microcirculatory resistance in multiple coronary artery regions may be particularly useful in patients with typical angina pectoris without obstructive coronary artery disease. However, the abnormality in cases of coronary microvascular dysfunction (CMVD)^20^ may not involve all coronary microvessels of a major coronary branch uniformly, but may instead be distributed in the myocardium in a scattered manner, with a sparse distribution of myocardial ischemia. In such situations, significant differences may exist in microvascular coronary resistance even among the three coronary branches. Therefore, measuring the IMR in multiple coronary territories may be useful, particularly in patients with typical angina in the absence of obstructive coronary artery disease. In addition, abnormalities in cases of CMVD are not consistently present in all coronary microvessels in the main coronary branches. Instead, these abnormalities are distributed unevenly in the local myocardium, causing myocardial ischemia to be sparsely scattered. Measuring microvascular resistance in multiple coronary artery territories is thus crucial in comprehending the extent of damage distribution.

In the present study, similar to investigations using continuous coronary thermodilution^21^ or positron emission tomography (PET),^22^, ^23^, ^24^ interindividual variability in CBF_QCA_ and hMVRI_QCA_ values was observed. The main factor explaining the large ranges in flow and resistance values appears to be the dependence on myocardial mass. PET‐derived flow and resistance express these measurements per unit of tissue mass (i.e., milliliters per minute per gram of tissue). Indeed, when adjusting CBF and microvascular resistance for mass, similar values were found for the three different myocardial territories within the same patients. Even so, even when adjusted for mass, inter‐individual values for CBF and microvascular resistance show a considerable range.^23^, ^24^ Further confirming the hypothesis of natural variation in hyperemic myocardial perfusion, rather than measurement inaccuracies, Everaars et al.^24^ found hyperemic flow values in the range of approximately 50–450 mL/min in arteries. The large ranges of hyperemic flow values also found in the present study thus seem likely to correspond to a naturally occurring large variability of normal hyperemic values.

## 5 STUDY LIMITATIONS

Several study limitations to this investigation should be acknowledged.

First, the present study was performed at a single facility and involved a relatively small population.

Second, the papaverine intracoronary method is less well validated than the adenosine method and the results may not be the same as with adenosine.

Third, methods of calculating flow using APV are susceptible to several potential sources of inaccuracy. APV depends on the assumptions that a time‐averaged parabolic flow velocity profile occurs in which peak velocity is twice the mean velocity, that the peak of the flow velocity profile remains within the sample volume of the transducer throughout the cardiac cycle, and that the flow velocity profile remains unchanged with changes in flow rate. Normal proximal coronary arteries have been shown to have variable flow velocity profiles, the shapes of which may be almost perfectly parabolic, skewed toward the inner or outer wall, or trapezoidal.^6^, ^25^, ^26^, ^27^, ^28^ Near branches and in vessels that are tortuous or have intimal irregularities or stenoses, significant flow separation and deformation of the flow field is encountered.^25^, ^29^ In irregular, branched or ectatic vessels, the relationship between peak and mean velocity is likely to be even more inconsistent. This may limit the usefulness of quantitative flow calculations based on APV, particularly in patients with diffuse coronary disease. Furthermore, unless accurate angiography is performed, inaccuracies in our estimations of vessel cross‐sectional area may have occurred and may have limited the reliability of the findings.

Fourth, the relationship between CBF and vessel diameter in the present study was not investigated using PET or other modalities.

## 6 CONCLUSION

Comparative evaluation of microvascular resistance in coronary branches of varying diameters requires that vessel diameter be accounted for.

## 7 CLINICAL PERSPECTIVES

### 7.1 Clinical competencies

The IMR is a quantitative and reproducible indicator of coronary microvascular function and is essential for diagnosing INOCA.

- The LAD is the preferred target vessel in many studies, reflecting myocardial mass and coronary arterial predominance, and much evidence for the assessment of physiology in INOCA has been measured using the LAD.
- The IMR calculated by APV was higher for the RCA than for the LAD.

### 7.2 Translational outlook

The IMR, taking into account vessel diameter, showed no significant differences between coronary branches.

- For near‐constant vessel diameter, CBF showed a very strong linear correlation with APV, with the slope of the line with APV decreasing as vessel diameter increased.

## AUTHOR CONTRIBUTIONS

**Takahiro Muroya**: conceptualization; data curation; formal analysis; investigation; methodology; project administration; resources; software; supervision; validation; visualization; writing—original draft; writing—review & editing. **Hiroaki Kawano**: conceptualization; data curation; formal analysis; investigation; methodology; project administration; supervision; validation; visualization; writing—review & editing. **Fumi Yamamoto**: investigation; project administration. **Koji Maemura**: project administration; supervision; validation. All authors have read and approved the final version of the manuscript.

## CONFLICT OF INTEREST STATEMENT

The authors declare no conflicts of interest.

## TRANSPARENCY STATEMENT

The lead author Takahiro Muroya affirms that this manuscript is an honest, accurate, and transparent account of the study being reported; that no important aspects of the study have been omitted; and that any discrepancies from the study as planned (and, if relevant, registered) have been explained.

## Data Availability

Author elects to not share data.
